# Altering our (soils’) future

**DOI:** 10.1038/s44319-026-00892-4

**Published:** 2026-07-31

**Authors:** Karsten Zengler

**Affiliations:** 1 Department of Pediatrics, University of California, San Diego, La Jolla, CA 92093 USA; 2 Department of Bioengineering, University of California, San Diego, La Jolla, CA 92093 USA; 3 Center for Microbiome Innovation, University of California, San Diego, La Jolla, CA 92093 USA; 4 Program in Materials Science and Engineering, University of California, San Diego, La Jolla, CA 92093 USA; 5 Soil Health Center, University of California, San Diego, La Jolla, CA 92093 USA; 6 Prosper Soils Institute, Walnut Creek, CA 94597 USA

**Keywords:** Biotechnology & Synthetic Biology, Evolution & Ecology, Microbiology, Virology & Host Pathogen Interaction

## Abstract

The soil microbiome plays a crucial role for crop yield and plant nutrient content, and for sequestering carbon dioxide from the atmosphere. Recent advances in soil engineering therefore have great potential for sustainable and regenerative agriculture while contributing to climate resilience.

**Figure:**
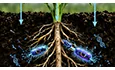

Soils cover only around 20% of the Earth’s surface, but this thin layer of organic and inorganic material produces about 95% of the world’s food supply for a growing human population of more than 8.1 billion people. Moreover, soils and the terrestrial ecosystems absorb one-third of CO_2_ emitted through natural and human activities (Bar-On et al, 2025). The dual role of soils as major food-producing and carbon-storing ecosystems highlights their eminent importance for humankind. Therefore, soil improvements through various soil alteration and engineering approaches have been on the forefront for both agricultural and climate mitigation efforts and represent the cornerstone for prosperity for the human race on a changing planet.

“Soils cover only around 20% of the Earth’s surface, but this thin layer of organic and inorganic material produces about 95% of the world’s food supply for a growing human population…”

Since ancient times, humans have implemented various methods to improve soil quality and boost plant growth: using manure as fertilizer or crop rotation, the habit of cultivating different plants sequentially, has been in use since about 6000 BC. While these ancient practices are still commonly used, the development of molecular and biological tools have now opened the door for more targeted soil engineering. Here, I will discuss the rocky path of soil engineering and the technical and economic challenges when deploying engineered or native microbes to the field, along with alternative efforts that could provide more tangible success in the near future.

For this article, I will refer to soil engineering as efforts to target single microbes or whole microbial communities to achieve a desired outcome, such as better plant performance, improved nutrient content or carbon sequestration. Traditionally, soil engineering has involved modifying only individual or a few organisms at a time. More recently, it has expanded to the entire microbial ecosystem. The approaches discussed here refer to genetic engineering of microbes, as well as directly modifying the soil microbiome through probiotics or prebiotics. Another approach is plant engineering to promote the growth and activity of specific microbes.

## The role of microbes

**Figure 1:**
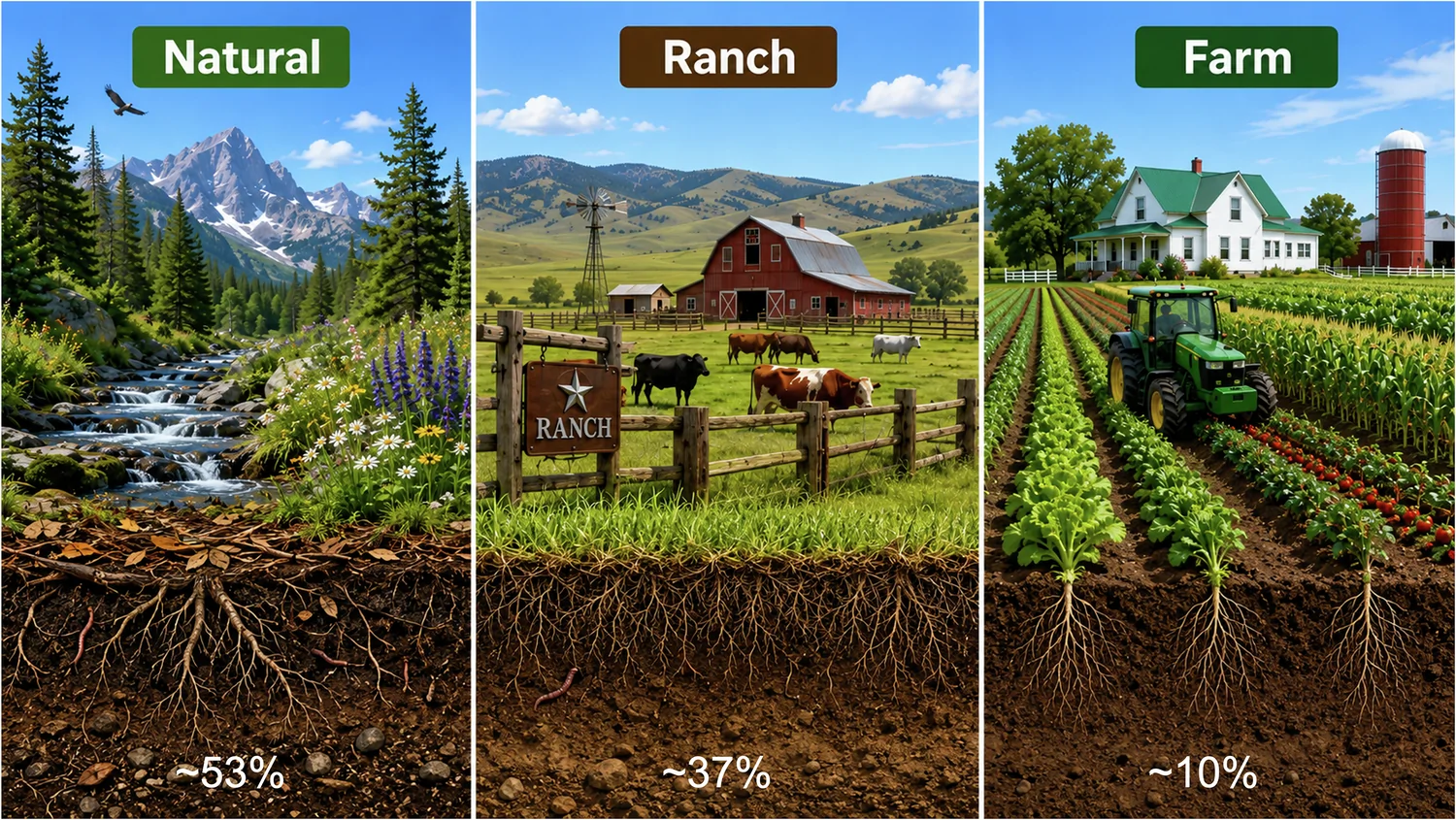
Breakdown of Earth’s habitable land use. Out of Earth’s total land mass, 25% are not habitable, being covered by ice, deserts, salt flats, or sand. Roughly half of the habitable land (47%) is used for agriculture, both for crop production (farmland), as well as for animal grazing (ranchland). Crops for direct human consumption make up around 16% and 4% are being used for textile and biofuel production, while the majority of agricultural land (80%) is currently used to grow crops for animal feed (source: https://ourworldindata.org/global-land-for-agriculture).

Around half of the world’s habitable land surface is already being used for agriculture (Fig. 1). This involves not only farmland for crop production but also a substantial proportion of ranchland. For example, 45% of all agriculturally used land in the USA is ranchland according to the USDA’s 2022 Census of Agriculture. Soil engineering solutions for sustainable agriculture or carbon sequestration should therefore ideally be suitable for all types of land, and not just farmland. So far, though, the major focus of soil alteration and engineering has traditionally been on farmland and particularly on improving crop yield and performance.

“Soil engineering solutions for sustainable agriculture or carbon sequestration should therefore ideally be suitable for all types of land, and not just farmland.”

The idea of using microbes to boost crop yield has been around for a long time: applying microbial inoculants, such as various rhizobia bacteria to enhance nitrogen fixation in leguminous plants, was first used in the late 18th century. While there are well-documented growth effects of *Rhizobium* species on legumes and other flowering plants, the effectiveness of microbial inoculants has often been limited by environmental factors, such as soil acidity, salinity or high nitrogen content of the soil (Abdissa Deberssa, 2022). As scientists identified the genetic basis for nitrogen fixation, it became possible to engineer *Rhizobium* species for specific field applications and environmental conditions (Riaz et al, 2025). Yet, even while *Rhizobium*-mediated growth promotion has been widely reported in legumes, the use of other microbial inoculants—native or engineered—for non-legume plants has been much less consistent (Hanif et al, 2024).

Another widely used bioinoculant are arbuscular mycorrhizal fungi (AMF), such as members of the *Glomeraceae* family. These soil fungi colonize the roots of about 80% of all terrestrial plants and form a symbiosis that benefits plant growth by enhancing phosphorus uptake, promoting drought resilience or protecting the plant against soil-borne pathogens (Delaeter et al, 2024). In addition to improving plant yield, microbes have also been shown to influence the overall nutrient content of plants, increasing their nutritional quality for humans (Abou Jaoudé et al, 2025). This is an active and exciting area of current research since improved nutrient content could provide improved food without the need for further increasing yield.

While alteration of soils often goes hand in hand with enhancement of plant growth or other plant-related features such as drought resistance, pathogen resilience or crop protection, there is also the opportunity for carbon capture and thereby counter increasing CO_2_ emissions (Beattie et al, 2025). In theory, this could be done not only on agricultural land—both farm- and ranchland—but also on wild habitats.

The idea behind carbon capture is to sequester CO_2_ from the atmosphere and convert it into recalcitrant carbon molecules that are not easily degraded by microbes and therefore accumulate in the soil. Such compounds are, for instance, highly branched or cross-linked polymers and aromatic compounds; instead of re-entering the carbon cycle, these molecules can persist in the soil over scales ranging from decades to millennia. Recalcitrant molecules can either be produced by the plant and exuded into the soil; indeed, up to 25% of all photosynthetically fixed carbon in plants is released into the rhizosphere through root exudates (Badri and Vivanco, 2009). Alternatively, recalcitrant carbon could be produced by rhizosphere microbes from plant exudates or necromass, that is, dead organisms. Another strategy to increase carbon capture by soils is to reduce microbial degradation of plant-derived recalcitrant compounds. Overall, an increase in soil carbon sequestration would contribute significantly to the global reduction of greenhouse gases without affecting food production.

“Overall, an increase in soil carbon sequestration would contribute significantly to global reduction of greenhouse gases without affecting food production.”

## Soil amendments

**Figure 2:**
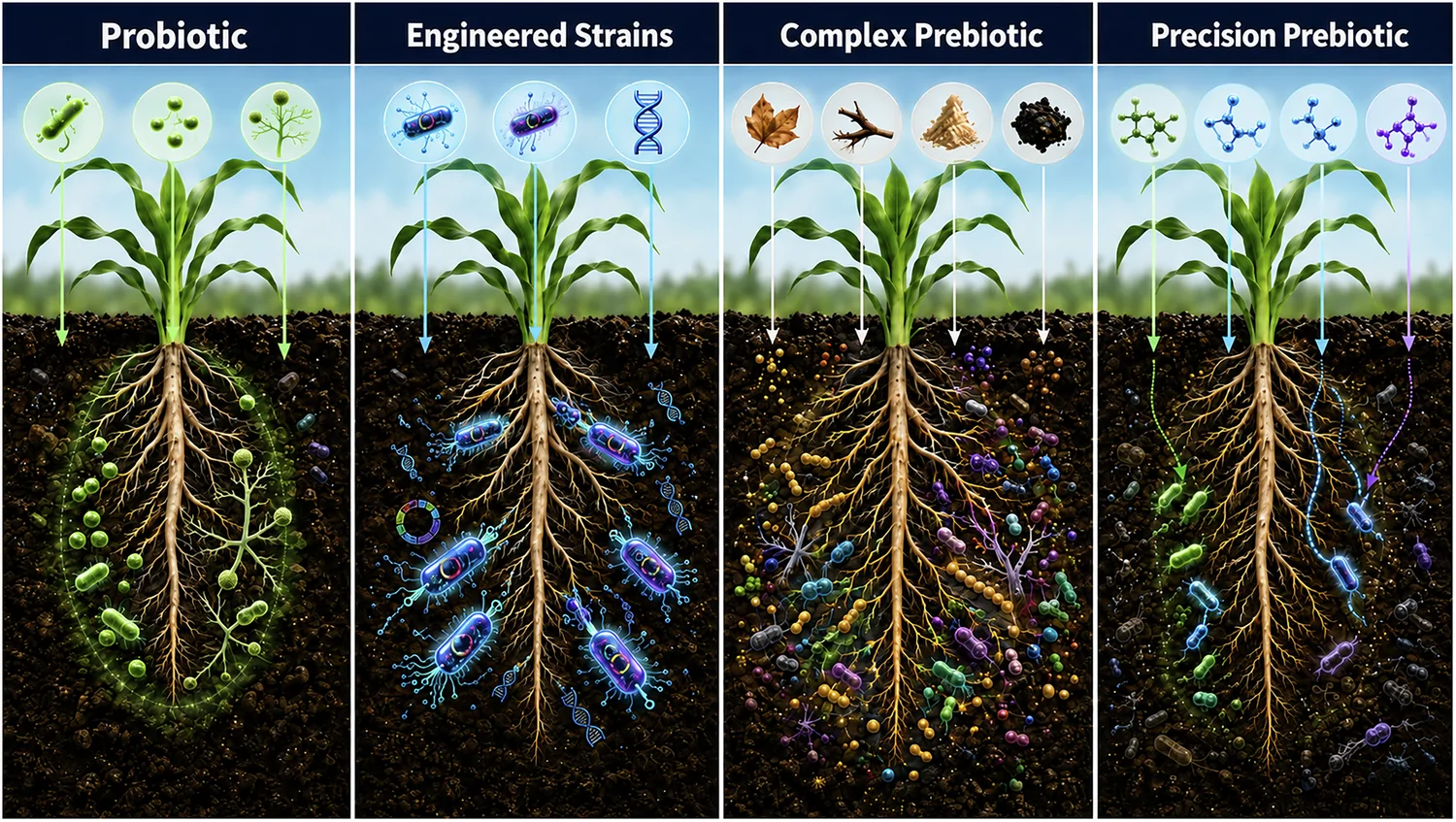
Overview of various soil amendments. We can distinguish between four different types of soil amendments; (i) probiotics (native or non-native soil bacteria), (ii) genetically engineered microbes, or alternatively genetically alterations of microbes in vivo, (iii) addition of complex organic and inorganic mixtures (i.e., prebiotics), such as compost or various waste products, and (iv) precision prebiotics, which are single compounds (or mixtures of a few selected compounds) intended to boost specific soil microorganisms.

Soils can be efficiently altered by adding organic material, or specific prebiotics or probiotics. Traditionally, farmers have treated farmlands by applying agricultural, human and industrial waste, compost, or other organic mixtures such as humic acids (Fig. 2) for millennia. Such soil amendments have shown both short- and long-term effects on carbon sequestration, as well as on crop yield and water retention (Kutos et al, 2023). Generally, these mixtures contain many different compounds and microbes that can be either beneficial or potentially harmful. Moreover, using natural organic mixtures, it is not possible to target microbe(s) in a way that they specifically influence plant performance, plant nutrient content or carbon sequestration. To achieve more specific results, it is necessary to manipulate soils and soil microbiomes in a more nuanced and tailored way that accounts for different types of soils, the plants and crops grown there, and various environmental factors, as well as seasonality.

In contrast to mixtures such as manure and compost, prebiotics—organic or inorganic additions—and probiotics—specific microbes—are such defined and nuanced soil amendments (Fig. 2). Prebiotics are intended to boost the growth of beneficial microorganisms that are present in the soil. But given the complexity of the soil microbiome, it is difficult to identify the ideal prebiotics for a given plant or soil situation. Probiotics, on the other hand, have been used to boost plant performance for more than a century, and many bacterial and fungal products are commercially available. These can include both wildtype as well as genetically engineered strains. While some probiotic treatments do improve plant performance, it is not clear if they achieve their effects directly or indirectly, which is difficult to validate without appropriate controls. For instance, many probiotic field trials do not include autoclaved or sterilized probiotics as a control (Averill et al, 2022). For instance, since dead bacterial biomass can serve as an organic fertilizer, it is crucial to distinguish between results obtained from living cells or dead cells (Thiruppathy et al, 2025).

## Genetic engineering

Engineering of soils, and soil microbes in particular, is often synonymous with synthetic biology (Way et al, 2014), an extension of what was referred to as metabolic engineering or bioengineering decades ago and before that as genetic engineering. So far, most applications of synthetic biology or genetic engineering in agriculture have focused on commercially important crops to improve yield, disease and virus resistance, herbicide tolerance, non-browning and shelf life, as well as to enhance environmental stress tolerance and nutritional content.

Yet, plant engineering methods developed for commercial crops are not easily applicable to rangeland or wild plants. In addition, not every plant-related issue—such as nitrogen availability—can be solved by genetically modifying the plant, but instead requires alteration of the microbes that are either symbiotic with the plant or play a crucial role in supporting plant growth. Thus, engineering of microbes—which is a more straightforward approach than plant engineering—is often an easier alternative (Fig. 2). While the tools to genetically modify microorganisms are manyfold and have been vastly improved over the years, there have not been major breakthroughs in terms of successfully deploying single-engineered microbes in the field. Even though engineered microbial strains are currently used in agriculture (Woodward et al, 2025), these products often face competition from chemical fertilizers that can be produced much more economically at scale.

“While the tools to genetically modify microorganisms are manyfold and have been vastly improved over the years, there have not been major breakthroughs in terms of successfully deploying single-engineered microbes in the field.”

Synthetic biology has been very successful for single-microbe applications, and there are many examples from biofuels to specialist chemicals (Winegar et al, 2025). Synthetic biology relies on a deep understanding of the microbe’s genetic and metabolic pathways or high-throughput screening methods to identify suitable strains for selection and subsequent improvement. Hence, synthetic biology to improve the production of a certain compound, works very well for biotechnological and industrial applications, where production takes place in highly controlled closed systems, such as bioreactors, where it can be readily validated and scaled. The capability to screen many variations in combination with well-established procedures to reduce technical and economic risks has contributed vastly to the success of synthetic biology (Winegar et al, 2025).

However, the principles of synthetic biology for single microbes are not readily transferable to complex microbial communities with many, often undefined members. Thus, the luxury of an in-depth understanding of the system, that is, a comprehensive genetic and biochemical knowledge of all individual microbes, to enable rationale engineering does not pertain to soils. While AI approaches are currently being developed to obtain such deeper knowledge of highly complex systems, there has not been a breakthrough reported yet that uses AI to effectively change soil microbiomes in a predictive manner (Gilbert and Zengler, 2025).

Natural environments, and soils in particular, are highly complex and diverse systems that are difficult to control and therefore rational designs targeting single microbial members routinely do not produce the desired outcomes. These failures can be broadly explained by two factors. First, lab experiments and models do not translate into field performance. One important reason is that results from single microbes or pairwise experiments do not reflect the situation of microbial communities (Moyne et al, 2026). It is therefore crucial to design studies that test complex communities—so-called synthetic microbial communities or SynComs—so that preliminary lab studies successfully translate to field studies where the microbes compete with other soil community members in a dynamic setting. Second, the chosen microbes or their engineered counterparts often do not engraft in the native environment. This issue is similar to probiotic applications in humans, for instance, when non-native *Lactobacillus* species are commonly used as a probiotic, but do not engraft readily in the human gut. Native bacteria used as soil probiotics could thereby overcome the engraftment challenge.

“Natural environments, and soils in particular, are highly complex and diverse systems that are difficult to control and therefore rational designs targeting single microbial members routinely do not produce the desired outcomes.”

When engineered native strains are used as a probiotic, it is also important to account for fitness. For example, strains engineered for improved nitrogen fixation might not perform well in a natural environment because nitrogen fixation is an energetically costly process (16 ATP per fixed N_2_), which might not be sustained in every soil type because of its substantial fitness cost to the organism.

Besides engineering bacteria in the laboratory, there is also the possibility to perform engineering of microbes in situ (Fig. 2): DART (DNA-editing all-in-one RNA-guided CRISPR–Cas transposase), for example, is a method that enables in situ engineering (Rubin et al, 2022). Still, again, it is currently unclear how to control engineering of one—and only one—specific member of a given microbial community without creating unwanted side effects. Another hurdle of in situ engineering is biocontainment: to contain the engineered microbes within their specific target environment (Arnolds et al, 2021). Yet, another daunting task in situ engineering faces is how to screen for outcomes at scale. It might be cost-prohibitive to perform screens of hundreds of field soils to evaluate the best outcomes and recover causative strains.

## Engineer the system, not the microbe

Deploying a synthetic biology approach for soil microbiome engineering has been a challenge, largely because we have a limited understanding of all the factors that control microbial diversity in such a diverse and dynamic system. I would therefore argue that instead of a “surgical” engineering approach focused on a single or a handful of microbes, it would be more beneficial to alter the entire system to change the state of the soil. When curing a complex human disease, multiple factors need to be changed to effectively move the system from a diseased to a healthy state. This normally does not require to eradicate every single pathogen; instead, it would reduce the number of pathogenic cells so that the immune system and commensal microbes can control pathogenic dysbiosis. Similar to the disease analogy, we could envision converting a non-productive depleted soil into a healthy soil by promoting certain microbes and disadvantaging others (Moyne et al, 2026). This could be done not by engineering one particular gene, pathway or microbe, but by changing the entire microbial and metabolic state of the soil. To do so, we require tools that accurately predict the outcome of a specific intervention in a complex system.

“I would therefore argue that instead of a “surgical” engineering approach focused on a single or a handful of microbes, it would be more beneficial to alter the entire system to change the state of the soil.”

To achieve this, we developed an approach called Microbial Interaction and Niche Determination (MIND; Moyne et al, 2026). MIND determines how microbes compete within complex communities and identifies their specific nutrient preferences, thereby delineating complex competitive interactions. Furthermore, it predicts how communities will respond when species (probiotics) or nutrients (prebiotics) are added, enabling predictive microbiome engineering. MIND worked for many applications, including in soil mesocosms, but also in live animals, such as a mouse model (Moyne et al, 2026). Combining MIND with computational modeling at the genome scale (Zaramela et al, 2021), we are now in a position to successfully design precision prebiotics: interventions that are specific for certain soil types and environments (Fig. 2). MIND can also assess the effect synthetic consortia will have on the native community, potentially preventing unwanted consequences on the native microbiome.

## Choosing the optimal approach

There are several factors to take into account when altering soil microbiomes to achieve desired outcomes. It is often easier to apply prebiotics to boost beneficial bacteria already residing in the soil (Moyne et al, 2026) than to introduce live probiotic bacteria. For example, live bacteria might not be easily grown, stabilized and formulated at scale, which is especially challenging for non-spore- forming, or strictly anaerobic microbes. Moreover, as discussed above, specific species or strains could fail to establish themselves in existing microbial communities. Additionally, probiotics—bacteria and fungi—are differently regulated by different countries, making it often challenging to generate viable products for a global market (Chandler et al, 2011). Prebiotics, on the other hand, are generally more cost-effective to produce and to apply. Furthermore, the industry benefits from a long experience in determining the optimal formulation for increased shelf life and field deployment.

Another consideration for finding the best method to alter soil microbial communities is the operational costs. Microbes have long been used for seed coating, which incurs little additional cost and does not change farming practices. However, if probiotics or engineered microbes need to be deployed during the growing season, eventually multiple times, the purchasing and operations costs could increase drastically. Given how much carbon (C), nitrogen (N) and phosphate (P) is needed to grow microbial products at scale, the same amount of C/N/P could be used directly as fertilizer with much less investments required. To make microbe deployment in the field-scale a cost-effective alternative to readily available fertilizers, the costs for production, safe storage, shipping and applying them have to be substantially less than the expected increase in crop yield and ideally should not require farmers to adopt new practices and routines.

“… if probiotics or engineered microbes need to be deployed during the growing season, eventually multiple times, the purchasing and operation costs could increase drastically.”

A more effective and economic approach would be to develop precision prebiotics for improving soil and plant health, or enhancing nutritional content (Moyne et al, 2026). Such prebiotics could also be applied for long-term carbon sequestration on farm and ranchland, as well as in wild habitats. However, the carbon balance for producing, shipping and deploying prebiotics compared to the achievable carbon sequestration needs to be carefully evaluated.

Overall, many advances have been made over millennia to alter soils and to improve crop yield, nutrient content, drought and pathogen resistance of plants and to sequester more carbon. These advances—from crop rotation and natural fertilizers to the green revolution and genetic engineering of plants and microbes—have enormously increased agricultural production and thereby provided the basis for population growth and the rise of civilization. Yet, given the challenges of climate change, environmental degradation and the need to feed an increasing human population, targeted soil engineering might well be the next step to ensure sustainable and regenerative agriculture and to contribute to climate resilience.

## Supplementary information

Peer Review File
