# Supplementary material for: Altering our (soils’) future
Source: EMBO Rep. 2026 Jul 31;27(16):4609–13. doi: 10.1038/s44319-026-00892-4 (PMC13503923; doi:10.1038/s44319-026-00892-4)
Supplement: Supplementary file 1 [file 44319_2026_892_MOESM1_ESM.pdf]

EMBO reports

Altering Our (Soils') Future

Karsten Zengler

Corresponding author(s): Karsten Zengler (kzengler@ucsd.edu)

Review Timeline:

Submission Date:	7th Jul 26
Accepted:	22nd Jul 26

Editor: Holger Breithaupt

Transaction Report: This article was editorially reviewed.
